# Sociodemographic Paradoxes and Enrollment Differences in In-Person Versus Online Recruitment to a Mobile Health Smoking Cessation Intervention for Food-Insecure Adults: Secondary Analysis of a Randomized Controlled Trial

**DOI:** 10.2196/80530

**Published:** 2026-06-11

**Authors:** Charles E Hoogland, Steven K Sutton, Sarah R Jones, Bence Nagy, Samuel J Brockway, David Himmelgreen, Thomas Mantz, Michael S Businelle, Ya-Chen Tina Shih, Jennifer I Vidrine, Damon J Vidrine

**Affiliations:** 1 Department of Health Outcomes & Behavior, Moffitt Cancer Center, 12902 USF Magnolia Drive, Tampa, FL, 33612, United States, 1 (813) 745-7937; 2 Department of Biostatistics and Bioinformatics, Moffitt Cancer Center, Tampa, FL, United States; 3 Department of Oncologic Sciences, Morsani College of Medicine, University of South Florida, Tampa, FL, United States; 4 Department of Psychology, University of South Florida, Tampa, FL, United States; 5 Department of Anthropology, Center for the Advancement of Food Security and Healthy Communities, University of South Florida, Tampa, FL, United States; 6 Feeding Tampa Bay, Tampa, FL, United States; 7 TSET Health Promotion Research Center, Stephenson Cancer Center, University of Oklahoma Health Sciences Center, Oklahoma City, OK, United States; 8 Department of Family and Preventive Medicine, University of Oklahoma Health Sciences Center, Oklahoma City, OK, United States; 9 Jonsson Comprehensive Cancer Center, Program in Cancer Health Economics Research, Department of Radiation Oncology, School of Medicine, UCLA Jonsson Comprehensive Cancer Center, Los Angeles, CA, United States

**Keywords:** recruitment strategies, smoking cessation, food insecurity, food assistance, randomized controlled trial, social status

## Abstract

**Background:**

Little is known about (1) sociodemographic, psychosocial, or smoking-related differences among individuals recruited to smoking cessation randomized controlled trials (RCTs) using in-person versus online recruitment methods or (2) the relative speed of recruitment using these 2 approaches. This secondary analysis is the first to examine these comparisons in a smoking cessation RCT for people experiencing food insecurity, a vulnerable special population for whom quitting is especially urgent.

**Objective:**

To compare (1) baseline sociodemographic, smoking-related, and psychosocial characteristics; and (2) screening, eligibility, and enrollment rates of in-person versus online recruits to a smoking cessation RCT for people experiencing food insecurity.

**Methods:**

Participants completed a brief eligibility questionnaire and a baseline assessment via tablet (in person) or personal electronic device (after clicking an online advertisement). Eligibility required past-30-day food aid use, smoking ≥5 cigarettes per day, and willingness to attempt quitting within 7 days post enrollment. Responses were compared using chi-squared and Fisher exact tests (categorical variables) and 2-tailed *t* tests (continuous variables).

**Results:**

Enrollees recruited online endorsed greater food insecurity (mean 4.5, SD 1.9 vs mean 3.0, SD 2.3; *P<*.001) and were more likely to be educated beyond high school or equivalent (69% vs 49%; *P*<.001), have household income of US $20,000 or more (46% vs 36%; *P*=.03), and be non-Hispanic White (77% vs 50%; *P<*.001). Online recruits indicated lower motivation to quit smoking (Contemplation Ladder; mean 7.2*,* SD 2.4 vs mean 8.0, SD 2.8; *P<*.001) and smoking cessation self-efficacy (mean 20.5, SD 8.0 vs mean 23.2, SD 8.6; *P<*.001). Online recruits also reported lower subjective social status (mean 4.6*,* SD 2.0 vs mean 5.9, SD 2.2; *P<*.001), greater financial strain (mean 17.9, SD 6.3 vs mean 16.2, SD 6.6; *P=*.004), more depressive symptoms (mean 8.6, SD 6.3 vs mean 7.4, SD 6.1; *P=*.04), greater loneliness (mean 6.0, SD 2.1 vs mean 5.2, SD 2.0; *P<*.001), less resilience (mean 19.5, SD 5.1 vs mean 20.5, SD 4.3; *P=*.02), less alcohol misuse (27% vs 37%; *P=*.02), and more past-30-day cannabis use (25% vs 15%; *P*=.01). Enrollment rates were higher online (64.8 per month; n=324) than in-person (7.7 per month; n=178). Although screened eligible at similar rates whether recruited online or in person (79% vs 75%; *P=*.10), eligible online individuals were more likely to enroll (71% vs 49%; *P<*.001).

**Conclusions:**

This study is the first to compare baseline participant characteristics by recruitment method (in person vs online) in a cessation RCT for people experiencing food insecurity and to evaluate the relative pace of recruitment via those methods. Online and in-person recruits were demographically and psychosocially distinct, and online recruitment was associated with faster accrual than in-person recruitment. These findings inform recruitment strategies for cessation interventions, especially those targeting food-insecure individuals.

## Introduction

### Background and Rationale

Cigarette smoking remains the leading cause of preventable disease and death in the United States [1]. Although the prevalence of smoking has been declining for decades, smoking rates have declined more slowly in socioeconomically disadvantaged groups [1,2]. One such group facing unique challenges in quitting smoking is people experiencing food insecurity or a lack of consistent access to sufficient food to lead a healthy, active life [3]. A person’s level of food security is largely determined by purchasing power, which is mostly derived from wages, government transfers (eg, Social Security payments), and household composition [4], and money spent on cigarettes becomes unavailable to be spent on essential goods, including food. Although food insecurity is associated with both financial strain and psychological distress, it is also an independent predictor of smoking status and intensity and likely impedes smoking cessation success via multiple mechanisms, such as the reinforcement of smoking via nicotine’s appetite suppressant effects and negative affect reduction [5]. Thus, there is a critical need to directly target individuals experiencing food insecurity with evidence-based smoking cessation interventions. In this population, successful long-term smoking cessation might be especially beneficial, as it may lead not only to abstinence-based health improvements but also to potential increases in food security due to both eliminating spending on cigarettes and reducing spending on smoking-related health care costs.

Recruiting special populations (eg, patients with cancer) to smoking cessation intervention trials can be very challenging, with eligibility percentages among those contacted sometimes in the low single digits [6]. Given these and other obstacles to obtaining desired sample sizes, researchers targeting various special populations have often used innovative recruitment strategies. For example, a recent feasibility trial for a text-based smoking cessation intervention for sexual and gender minorities yielded 79 participants following the posting of approximately 700 flyers in a variety of public establishments in the local catchment area, 1500 emails sent to potentially eligible participants on ResearchMatch (who were overwhelmingly from outside the catchment area), and the posting of advertisements on Facebook, Instagram, and Craigslist. The screening yield rate for each modality was below 6%, although the enrollment rate among those screened was approximately 50% [7].

Latino Americans are another special population that can be difficult to successfully reach and enroll in smoking cessation randomized controlled trial (RCTs), necessitating multiple types of recruitment approaches. For example, a secondary analysis of data from *Decídetexto*, a mobile health smoking cessation RCT for Latino Americans conducted across 4 states, compared the effectiveness of outbound, direct recruitment strategies, in which study staff contacted potential participants, to inbound, mass recruitment strategies (eg, flyers and television, newspaper, or Facebook advertisements), in which interested individuals had to contact study personnel to be screened [8]. Although most enrollees were recruited via “high effort” direct strategies (eg, in-person community outreach or personal phone calls to patients in registries), individuals recruited through either mass recruitment or “low effort” direct strategies (eg, emailing or texting patients in registries) were more likely to screen eligible and to enroll in the trial. In terms of personal characteristics, mass-recruited enrollees differed considerably from enrollees recruited via direct strategies, including having lower socioeconomic status, being less likely to have health insurance, and being less acculturated (eg, being more likely to primarily speak Spanish).

A contrasting pattern emerged from a smoking cessation RCT conducted by our team for another special population, cervical cancer and precancer survivors [9,10]. Enrollees recruited online (vs enrollees recruited from clinics at a National Cancer Institute–designated cancer center) had higher levels of education and health literacy but did not differ in age, race, household income, smoking behaviors, or motivation to quit smoking [9]. Thus, online recruitment strategies (eg, social media advertisements) cannot be presumed to consistently yield more- or less-disadvantaged members of special populations than in-person strategies.

To date, few studies have specifically addressed the process of recruiting people who smoke and are experiencing food insecurity to smoking cessation RCTs. One notable exception, however, was a pilot study that supported the feasibility of in-person recruitment of such individuals at food pantries to a smoking cessation treatment study [11]. The study entailed 22 research staff visits distributed across 4 food pantries in the Greater Cleveland region. During each site visit, the research staff set up an outreach table and remained there for between 1.5 and 3.5 hours, during which approximately 28% of the clients (173 of an estimated 628 total pantry visitors) approached their table. Of them, 31% (54/173) expressed interest in information on quitting smoking, and of those interested in information on quitting, 78% (42/54) were able to be contacted and screened for eligibility via phone. Ultimately, 18% (31/173) of eligible individuals gave consent to have their contact information sent to the Ohio Tobacco Quitline (ie, 5% [31/628] of individuals served by the food pantries during recruitment) [11]. Thus, the recruitment yield was low.

With the limited exceptions of the studies described earlier that recruited Latino Americans and cervical cancer or precancer survivors [8,9], few studies have examined (1) potential sociodemographic, psychosocial, or smoking-related differences among individuals recruited to smoking cessation trials using in-person versus online recruitment methods or (2) differences in recruitment yield and enrollment outcomes associated with these 2 methods [6,9]. Indeed, to our knowledge, the current secondary analysis represents the first such comparison using data from a smoking cessation RCT for people experiencing food insecurity, a vulnerable special population for whom quitting is especially urgent.

### Objectives

The primary objective of this secondary analysis was to compare baseline sociodemographic, smoking-related, and psychosocial characteristics of participants recruited via in-person versus online methods to a National Cancer Institute–funded smoking cessation trial evaluating the efficacy of a mobile health intervention for individuals experiencing food insecurity, as such differences may have clinically meaningful implications for trial design and outcome interpretation. A secondary objective was to describe screening, eligibility, and enrollment rates among individuals recruited in person versus online.

## Methods

### Trial Design

The study underlying this secondary analysis is a smoking cessation RCT evaluating the efficacy of a fully automated smartphone-delivered intervention (automated treatment [AT]) versus connections to state quitlines (standard treatment [ST]).

### Trial Setting

Individuals reporting current smoking and recent receipt of food assistance (eg, food bank or food pantry or electronic benefits transfer [EBT] or food stamps) were recruited in person (March 2022 to February 2024) and online (February 2024 to June 2024). All assessments were conducted online via REDCap (Research Electronic Data Capture), and participants were followed for 12 months [12]. Additional procedural information has been published previously [13].

### Intervention and Comparator

The AT intervention was fully automated after enrollment, whereas each ST participant’s contact information was provided to the appropriate state quitline, and the quitline proactively contacted the participant. All participants received a 10-week supply of combination nicotine replacement therapy (ie, nicotine patches and lozenges).

#### Outcomes

This secondary analysis focuses on participant recruitment via online and in-person modalities; however, the primary and secondary outcomes of the parent RCT are described here for context. The primary outcome of the RCT was self-reported smoking abstinence (missing=smoking) at the 12-month follow-up. The secondary outcomes included abstinence verified biochemically via salivary cotinine samples and self-reported continuous, 30-day, and 24-hour abstinence, all at the 12-month follow-up. For each outcome in the parent RCT, the proportion of participants abstinent in AT versus ST was compared. These outcomes are not reported in this secondary analysis.

#### Sample Size

The target sample size was 500 (250 per group), as that was estimated to provide 80% power to detect an increase in the self-reported smoking abstinence rate (missing=smoking) at 12 months of 7% in AT (vs ST) [13].

#### Randomization

After consenting to participate and completing the baseline assessment using REDCap, participants were randomly assigned to AT or ST following stratification on sex and nicotine dependence. Allocation was implemented via REDCap’s randomization module, and enrolling staff did not have access to the allocation sequence (see [13]).

#### Blinding

Participants were not blind to treatment condition.

#### Patient and Public Involvement

In-person recruitment was facilitated via a partnership with Feeding Tampa Bay, which aided the researchers in the conduct of the trial. There was no other public or patient involvement in the design, conduct, and reporting of the trial.

#### Changes to Trial Protocol

To increase recruitment, the study team collaborated with an advertising partner to develop an online advertising campaign (eg, online advertisements and the study landing page), which used targeted social media (such as Facebook and Instagram) and search engine advertising and ran for approximately 5 months, from February to June 2024.

#### Harms

Although this was a low-risk smoking cessation RCT, participant safety was systematically monitored in accordance with the study’s data and safety monitoring plan by the study multiple principal investigators (JIV and DJV) throughout the trial, with adverse events being defined as any unexpected physical, psychological, or social harms occurring during the trial period.

### Data Collection and Management

The AT participants used a unique link sent to their email address to download the treatment app. Data collected on the app were stored via a backend database meeting all Moffitt Cancer Center data security policies and procedures. Participants in the ST condition were connected to state quitlines that were free of charge to callers. All participants were assigned unique identification numbers for use in data analyses and for data transfer, in the event of a request. Only study identification numbers were used in analysis files.

### Participant Recruitment Strategies

#### In-Person Recruitment

Trained research coordinators, who generally worked in teams of 2 to 4, made 246 recruitment visits to food distribution events held at 86 sites throughout West Central Florida for 23 months (March 2022 to February 2024). The research coordinators wore matching, study-branded clothing and approached clients waiting in their automobiles (at events where clients received their food in their trunk) or sat at an event table and talked with food bank clients who approached the table (at the events where food bank clients were handed boxes of food or served meals in a restaurant-type setting).

#### Online Recruitment

The online recruitment targeting parameters were as follows: nationwide, smoking ≥5 cigarettes per day, having received food assistance in the past 3 months, being ≥18 years old, speaking English or Spanish, and not currently pregnant. Creation of the advertising partner’s proprietary advertisement targeting algorithm was based on machine learning and data mining using aggregated data sets from social media, online searches, health websites, and online patient communities. Advertisements did not change during the course of the nationwide campaign. Although optimization services were available in the event that recruitment targets were not being met, they were not used because participant accrual was relatively rapid throughout the online campaign. In the third month of the campaign (April 2024), the campaign budget was lowered by half to decrease the rate at which potential participants were recruited, an action that was taken to preserve staff bandwidth. Recruitment ended in June 2024, shortly after reaching the study’s target sample size (ie, 500 enrolled participants).

### Materials and Measures

#### Eligibility Criteria

Potential participants completed an electronic screener to determine eligibility. Tablets were used to administer the questionnaire to individuals approached in person. Primary eligibility requirements included receiving food assistance in the past 30 days (food bank, EBT, or food aid from a family member or friend), smoking ≥5 cigarettes per day, and willingness to attempt quitting within 7 days of enrollment [13].

#### Baseline Assessment

Participants indicated their degree of food insecurity by completing the 6-item short form version of the United States Department of Agriculture’s US Household Food Security Survey (eg, “In the last 12 months, did you ever eat less than you felt you should because there wasn’t enough money for food?”) [14]. Scores ranged from 0 to 6, with higher scores indicating greater food insecurity [14]. Other participant characteristics measured at baseline included sociodemographic, smoking-related, and psychosocial variables [13].

### Statistical Methods

Recruitment groups were compared on categorical variables using chi-squared and Fisher exact tests and on continuous variables using independent samples *t* tests. Effect sizes were gauged using Cohen *d* for continuous variables and either Cramer *V* or odds ratios for categorical variables. CIs for Cohen *d* were created using the noncentral *t* distribution method, creating exact interval estimates for the standardized mean differences [15], while 95% CIs for Cramer *V*, which is bounded between 0 and 1 and whose magnitude can be interpreted similarly to a Pearson correlation coefficient [16,17], were constructed using a nonparametric percentile bootstrapping procedure with 10,000 resamples [18-20]. The 95% CIs for the odds ratios were calculated using the standard, natural logarithm transformation–based formula [21].

To explore whether differences in self-reported food insecurity between the in-person and online groups might have been a consequence of basic demographic differences between the groups, a hierarchical linear regression analysis was performed. Little’s MCAR (missing completely at random) test was used to ascertain whether the data were MCAR [22]. Although there were no missing data on in-person versus online group membership, nor on food insecurity, cases with missing data on any of the predictors present in one or both models tested were deleted listwise, which resulted in a nominal 5% reduction of the analytic sample size from 502 to 475. To identify model covariates, univariate demographic predictors of either food insecurity (*P*<.10) or recruitment modality (*P*<.05) were identified using simple logistic (categorical variables) or linear (continuous variables) regression analyses [9]. (Candidate covariates included age, ethnicity or race, sex, sexual orientation, relationship status, educational attainment, health insurance coverage, household income, and employment status.) Next, variables meeting those criteria were entered as a set to estimate a covariates-only model (model 1). Before model inclusion, categorical variables were dichotomized (eg, income was approximately median split to form less than US $20,000 per year and US $20,000 or more per year categories) to prevent estimation problems (eg, coefficient instability and inflated SEs) [23]. The next step entailed adding recruitment modality to the model as a predictor (model 2). Differences in model fit were examined using an *F* test; a significant difference would indicate an improvement in model fit, meaning that there was an association between food insecurity and recruitment modality even after accounting for the effects of relevant demographic covariates. The 95% CIs for the proportion of variance in self-reported food insecurity explained by models 1 and 2 (ie, model *R*^2^) were based on a noncentral *F* distribution and created using R package MBESS’ function “ci.R2” with parameter Random.Predictors set to “FALSE,” as the model predictors were fixed, rather than random [15,24]. To generate the increment in *R*^2^ between models 1 and 2 (ie, Δ*R*^2^), nonparametric bootstrapping with 10,000 resamples was used. An inspection of the histogram of the bootstrapped Δ*R*^2^ estimates revealed slight positive skew in the distribution, and so both percentile and bias corrected and accelerated (BCa) bootstrap CIs were calculated and reported [20] (for details, see Multimedia Appendix 1).

To compare differences in enrollment patterns between participants recruited in person versus online, the flow of potential participants between prescreening and full enrollment in the trial was presented, keeping the in-person and online recruitment denominators (and thus percentages) as comparable as possible within the constraints of the available data. Prescreening numbers were defined as the number of people assessed for eligibility after being approached at food pantry sites and the number of people who were assessed for eligibility after clicking online advertisements. Regardless of whether they were recruited in person or online, potential participants who were not excluded at the prescreening stage completed the study’s eligibility questionnaire.

Potential participants who prescreened eligible (eg, aged ≥18 y, self-reported smoking, and receiving food assistance) were considered “unreachable” after not being able to be contacted following approximately 2 weeks of staff outreach attempts using a structured outreach schedule. For the first week following prescreening, 3 REDCap notifications were sent via email and text (using Twilio integration) to remind potential participants of the screening survey, and study staff attempted contact via phone, text, or email 3 to 5 times. The second week consisted of 3 contact attempts approximately every other day. A similar procedure was followed at other steps of the enrollment process. For example, a participant who consented to participate but did not complete the baseline questionnaire was considered unreachable after 2 weeks of contact attempts.

Next, those who screened eligible and were not excluded (either because they were unreachable or because they declined to participate) completed the informed consent process. The onboarding completion rate was defined as the number of people who enrolled in the trial divided by the number of people who completed the consent process. Enrollment rates for those who both consented to participate and subsequently completed the preenrollment baseline questionnaire were calculated by dividing the number of people who completed the baseline questionnaire by the number of people who enrolled in the trial.

To provide a fuller sense of the intensity of recruitment efforts by recruitment modality, time (in days) from screening completion to enrollment was reported, along with the number of contact attempts made prior to enrollment for online recruits. Owing to the positive skew in the distributions of these values, medians and IQRs were reported in addition to means and SDs.

### Ethical Considerations

#### Ethical Approval and Informed Consent

The RCT was approved by Advarra, which serves as Moffitt Cancer Center’s institutional review board (IRB 00000971). The IRB and the informed consent document allowed this secondary analysis without additional consent. Informed consent was obtained from all participants. A waiver of written documentation of informed consent was obtained, allowing potential enrollees recruited in person to verbally consent, while potential participants recruited online read the consent document themselves via a consent screen on REDCap and acknowledged consent without needing to sign electronically.

#### Privacy and Confidentiality

All data were securely stored and managed at Moffitt Cancer Center using REDCap, with access limited to authorized study personnel. No identification of individual participants or users is possible in any images of this manuscript or supplementary material.

#### Compensation

No compensation was provided before enrollment in the trial (eg, individuals were not compensated for completing the screening questionnaire). Potential participants recruited in person and online were made aware of the study’s compensation structure during the consent process and received identical compensation for all study-related tasks. Over the course of the 12-month study period, participants could potentially earn up to a total of US $510 [13].

### Other Information

#### Reporting Guidelines

The trial was reported in accordance with the CONSORT (Consolidated Standards of Reporting Trials) 2025 statement (Checklist 1) [25] and the CONSORT-EHEALTH (Consolidated Standards of Reporting Trials of Electronic and Mobile Health Applications and Online Telehealth) checklist (Checklist 2) [26].

#### Trial Oversight

The study's multiple principal investigators were responsible for compliance with all federal and institutional IRB policies and the study’s data and safety monitoring plan.

## Results

### Baseline Data

**Figure 1.**
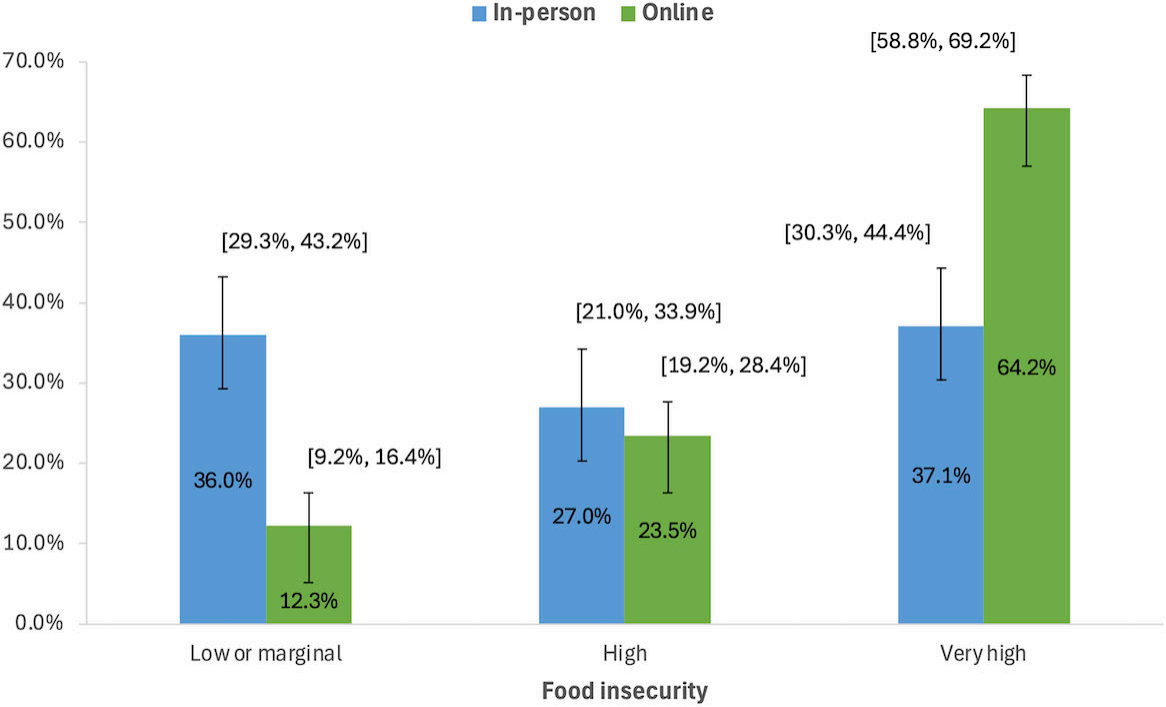
Secondary analysis of data from a mobile health smoking cessation randomized controlled trial for people with food insecurity: food insecurity by recruitment modality. Note: Error bars are 95% Wilson CIs.

Online (vs in person) recruits reported greater food insecurity (mean 4.5, SD 1.9 vs mean 3.0, SD 2.3; *d*=0.74, 95% CI 0.55-0.93; *t*_500_=7.93; *P*<.001). As illustrated in Figure 1, 64% (n=208) recruited online (vs in person: n=66, 37%) were classified as having very high food insecurity (odds ratio [OR] 3.04, 95% CI 2.05-4.53; *χ*^2^_1_=34.1; *P*<.001). Online recruits were also younger (45.7 vs 52.9 y; *d*=−0.70, 95% CI −0.89 to −0.51; *t*_500_=−7.48; *P*<.001) and less likely to be Hispanic or Latino (n=22, 7% vs n=39, 22%; OR 0.25, 95% CI 0.14-0.47; *χ*^2^_1_=24.9; *P*<.001). Participants recruited online were more likely to be female (n=250, 77% vs n=117, 66%; OR 1.76, 95% CI 1.15-2.69; *χ*^2^_1_=7.6; *P*=.006), non-Hispanic White (n=249, 77% vs n=88, 50%; OR 3.35, 95% CI 2.26-5.06; *χ*^2^_1_=38.3; *P*<.001), and LGBT+ (n=57, 18% vs n=11, 6%; OR 3.20, 95% CI 1.60-6.96; *χ*^2^_1_=12.5; *P*<.001). Moreover, online recruits were more likely to be educated beyond high school or a general educational development certificate (n=225, 69% vs n=87, 49%; OR 2.37, 95% CI 1.60-3.53; *χ*^2^_1_=20.7; *P*<.001) and to have annual household income of US $20,000 or more (n=150, 46% vs n=64, 36%; OR 1.52, 95% CI 1.03-2.26; *χ*^2^_1_=4.8; *P*=.03).

Online recruits reported lower motivation to quit smoking (Contemplation Ladder; mean 7.2, SD 2.4 vs mean 8.0*,* SD 2.8; *d*=−0.315, 95% CI −0.499 to −0.131; *t*_500_=3.38*; P<*.001), smoking cessation self-efficacy (mean 20.5, SD 8.0 vs mean 23.2, SD 8.6; *d*=−0.318, 95% CI −0.502 to −0.134; *t*_499_=3.40; *P<*.001), and subjective social status (mean 4.6, SD 2.0 vs mean 5.9, SD 2.2; *d*=−0.63, 95% CI −0.82 to −0.44; *t*_498_=−6.75; *P*<.001). They also reported more financial strain (mean 17.9, SD 6.3 vs mean 16.2, SD 6.6; *d*=0.27, 95% CI 0.09-0.46; *t*_486_=2.89; *P*=.004), depressive symptoms (mean 8.6*,* SD 6.3 vs mean 7.4*,* SD 6.1; *d*=0.19, 95% CI 0.01-0.38; *t*_498_=2.07; *P*=.04), and loneliness (mean 6.0, SD 2.1 vs mean 5.2, SD 2.0; *d*=0.40, 95% CI 0.22-0.59; *t*_499_=4.33; *P*<.001), as well as lower resilience (mean 19.5, SD 5.1 vs mean 20.5, SD 4.3; *d*=−0.22, 95% CI −0.40 to −0.03; *t*_493_=−2.30; *P*=.02).

Results were mixed with respect to substance use. Online recruits reported lower levels of past-year alcohol misuse (ie, binge drinking or heavy drinking, per National Institute on Alcohol Abuse and Alcoholism guidelines [27]; n=88, 27% vs n=66, 37%; OR 0.63, 95% CI 0.42-0.95; *χ*^2^_1_=5.3; *P*=.02). However, online participants were more likely to report having used one or more recreational drugs on a monthly or more frequent basis in the past year (n=74, 23% versus n=17, 10%; OR 2.78, 95% CI 1.56-5.22; *χ*^2^_1_=13.5; *P*<.001) and to report recreational cannabis use in particular within the past 30 days (n=80, 25% vs n=27, 15%; OR 1.82, 95% CI 1.10-3.07; *χ*^2^_1_=6.1; *P*=.01).

Further results (eg, on tobacco product use) are available in Table 1. Notably, using the Benjamini-Hochberg procedure [28,29] to control the false discovery rate (or FDR) by limiting the proportion of the significant analyses in Table 1 due to false positives to 0.05, only 3 (11%) of the 28 (out of 39 total) significance tests became nonsignificant. Specifically, differences between recruitment groups on typical cigarette type (regular vs menthol), age of smoking initiation, and depressive symptoms had *P* values <.05 but were nonsignificant according to the FDR-adjusted results.

**Table 1.** Secondary analysis of data from a mobile health smoking cessation randomized controlled trial for people with food insecurity: participant characteristics at baseline by recruitment modality.

Participant characteristics	Overall	Recruitment modality		*P* value^a^	Cohen *d* (95% CI) or Cramer *V* (95% CI)^b^
		In person	Online		
Food insecurity score (0‐6) (n=502), mean (SD)	3.9 (2.2)	3.0 (2.3)	4.5 (1.9)	<.001	−0.740 (−0.928 to −0.551)
Food insecurity group (n=502), n (%)				<.001	0.306 (0.222 to 0.394)
Low or marginal food insecurity (0‐1)	104 (21)	64 (36)	40 (12)		
High food insecurity (2-4)	124 (25)	48 (27)	76 (23)		
Very high food insecurity (5-6)	274 (55)	66 (37)	208 (64)		
Age (n=502), mean (SD)	48.2 (10.9)	52.9 (11.7)	45.7 (9.5)	<.001	0.698 (0.510 to 0.886)
Ethnicity or race (n=501), n (%)				<.001	0.336 (0.257 to 0.426)
Non-Hispanic White	337 (67)	88 (50)	249 (77)		
Non-Hispanic Black or African American	71 (14)	43 (24)	28 (9)		
Non-Hispanic mixed race or other	32 (6)	7 (4)	25 (8)		
Hispanic or Latino	61 (12)	39 (22)	22 (7)		
Sex (n=502), n (%)				.006	0.123 (0.034 to 0.213)
Male	135 (27)	61 (34)	74 (23)		
Female	367 (73)	117 (66)	250 (77)		
Gender identity (n=501), n (%)				.025	0.119 (0.046 to 0.212)
Male	137 (27)	61 (34)	76 (23)		
Female	360 (72)	115 (65)	245 (76)		
Transgender	4 (<1)	1 (<1)	3 (<1)		
Sexual orientation (n=500), n (%)				<.001	0.171 (0.099 to 0.248)
Straight or heterosexual	424 (85)	160 (91)	264 (81)		
Gay or lesbian, bisexual, or prefer to self-describe	68 (14)	11 (6)	57 (18)		
Prefer not to say	8 (2)	5 (3)	3 (<1)		
Relationship status (n=500), n (%)				.85	0.025 (0.011 to 0.130)
Single	203 (41)	71 (40)	132 (41)		
Married, living with significant other, or partnered	183 (37)	68 (38)	115 (36)		
Divorced, widowed, or separated	114 (23)	39 (22)	75 (23)		
Educational attainment (n=502), n (%)				<.001	0.207 (0.132 to 0.299)
Less than high school	48 (10)	25 (14)	23 (7)		
High school diploma or GED^c^	142 (28)	66 (37)	76 (23)		
Some college or vocational training or associate degree	256 (51)	73 (41)	183 (56)		
Four-year degree or more	56 (11)	14 (8)	42 (13)		
Health insurance coverage (n=479), n (%)				.003	0.135 (0.042 to 0.227)
Yes	400 (84)	128 (77)	272 (87)		
No	79 (16)	39 (23)	40 (13)		
Household income (US$; n=501), n (%)				.001	0.189 (0.121 to 0.283)
<10,000	139 (28)	58 (33)	81 (25)		
10,000-19,999	130 (26)	43 (24)	87 (27)		
20,000-29,999	89 (18)	34 (19)	55 (17)		
≥30,000	125 (25)	30 (17)	95 (29)		
Refuse to answer	18 (4)	12 (7)	6 (2)		
Employment status (n=502), n (%)				.55	0.065 (0.030 to 0.170)
Employed for wages or self-employed	153 (30)	53 (30)	100 (31)		
Out of work	96 (19)	29 (16)	67 (21)		
Homemaker, student, retired, or refuse to answer	119 (24)	47 (26)	72 (22)		
Unable to work	134 (27)	49 (28)	85 (26)		
Lives in household with another person who smokes (n=500), n (%)				.83	0.010 (0.001 to 0.102)
Yes	185 (37)	64 (36)	121 (37)		
No	315 (63)	112 (64)	203 (63)		
Lifetime quit attempts (n=494), n (%)				.41	0.077 (0.034 to 0.184)
0	49 (10)	18 (11)	31 (10)		
1‐4	268 (54)	84 (49)	184 (57)		
5‐8	76 (15)	29 (17)	47 (15)		
≥9	101 (20)	40 (23)	61 (19)		
Abstinence goal (n=499), n (%)				.20	0.109 (0.063 to 0.207)
Total abstinence, never use again	335 (67)	130 (73)	205 (64)		
Total, but could slip and maintain abstinence	81 (16)	24 (13)	57 (18)		
Occasional use when urges strongly felt	43 (9)	13 (7)	30 (9)		
Temporary abstinence	12 (2)	5 (3)	7 (2)		
No goal of abstinence at this time	28 (6)	6 (3)	22 (7)		
Cigarette type (n=501), n (%)				.04	0.090 (0.010 to 0.180)
Nonmenthol	271 (54)	85 (48)	186 (57)		
Menthol	230 (46)	92 (52)	138 (43)		
Other tobacco products used in past month (n=502), n (%)					
None	404 (80)	127 (71)	277 (85)	<.001	0.171 (0.080 to 0.262)
Cigars	43 (9)	27 (15)	16 (5)	<.001	0.175 (0.082 to 0.266)
Little cigars, cigarillos, bidis, or Black and Milds	57 (11)	21 (12)	36 (11)	.82	0.010 (0.001 to 0.104)
Pipe with tobacco	6 (1)	1 (<1)	5 (2)	.43	0.043 (0.003 to 0.098)
Dip, chew, or snus	5 (1)	3 (2)	2 (<1)	.35	0.051 (0.002 to 0.137)
Ever used e-cigarettes or vaped (n=501), n (%)				<.001	0.215 (0.128 to 0.305)
Yes	332 (66)	93 (53)	239 (74)		
No	169 (34)	84 (47)	85 (26)		
Last time used e-cigarettes or vaped (n=331), n (%)				.008	0.146 (0.044 to 0.248)
Within the past 30 d	134 (40)	27 (29)	107 (45)		
More than 30 d ago	197 (60)	66 (71)	131 (55)		
Cigarettes per day (n=502), n (%)				.11	0.111 (0.050 to 0.212)
0‐10	133 (26)	51 (29)	82 (25)		
11‐20	240 (48)	77 (43)	163 (50)		
21‐30	97 (19)	33 (19)	64 (20)		
≥31	32 (6)	17 (10)	15 (5)		
Time to first cigarette after waking (n=502), n (%)				<.001	0.222 (0.143 to 0.319)
After 60 min	29 (6)	17 (10)	12 (4)		
31-60 min	43 (9)	26 (15)	17 (5)		
6-30 min	195 (39)	70 (39)	125 (39)		
Within 5 min	235 (47)	65 (37)	170 (52)		
Heaviness of Smoking Index score (0‐6) (n=502), mean (SD)	3.3 (1.4)	3.1 (1.5)	3.4 (1.3)	.01	−0.233 (−0.416 to −0.049)
Age initiated smoking (n=492), mean (SD)	17.6 (6.2)	18.4 (7.2)	17.2 (5.6)	.04	0.199 (0.014 to 0.385)
Years smoking (n=502), mean (SD)	27.6 (11.6)	30.3 (12.9)	26.2 (10.6)	<.001	0.355 (0.171 to 0.539)
Contemplation Ladder (0‐10) (n=502), mean (SD)	7.5 (2.6)	8.0 (2.8)	7.2 (2.4)	<.001	0.315 (0.131 to 0.499)
Smoking cessation self-efficacy (9-45) (n=501), mean (SD)	21.5 (8.3)	23.2 (8.6)	20.5 (8.0)	<.001	0.318 (0.134 to 0.502)
Subjective social status (1-10) (n=500), mean (SD)	5.1 (2.2)	5.9 (2.2)	4.6 (2.0)	<.001	0.632 (0.444 to 0.820)
Financial strain (0‐26) (n=488), mean (SD)	17.3 (6.4)	16.2 (6.6)	17.9 (6.3)	.004	−0.275 (−0.462 to −0.088)
Depressive symptoms (0‐24) (n=500), mean (SD)	8.2 (6.2)	7.4 (6.1)	8.6 (6.3)	.04	−0.194 (−0.378 to −0.010)
Perceived stress (4-20) (n=497), mean (SD)	10.7 (3.1)	10.5 (2.8)	10.9 (3.2)	.19	−0.124 (−0.308 to 0.060)
Resilience (5-30) (n=495), mean (SD)	19.8 (4.9)	20.5 (4.3)	19.5 (5.1)	.02	0.216 (0.031 to 0.401)
Sense of control (8-32) (n=493), mean (SD)	23.9 (3.0)	23.6 (2.6)	24.1 (3.2)	.15	−0.135 (−0.319 to 0.050)
Loneliness (3-9) (n=501), mean (SD)	5.7 (2.1)	5.2 (2.0)	6.0 (2.1)	<.001	−0.405 (−0.589 to −0.220)
Alcohol misuse, past year (n=499), n (%)				.02	0.103 (0.017 to 0.194)
Yes	154 (31)	66 (37)	88 (27)		
No	345 (69)	111 (63)	234 (73)		
Recreational drug use frequency, past year (n=501), n (%)				.004	0.175 (0.118 to 0.257)
Daily or almost daily	56 (11)	9 (5)	47 (15)		
Weekly	19 (4)	3 (2)	16 (5)		
Monthly	16 (3)	5 (3)	11 (3)		
Once or twice	73 (15)	31 (18)	42 (13)		
Never	337 (67)	129 (73)	208 (64)		
Recreational cannabis use, past 30 days (n=501), n (%)				.01	0.110 (0.026 to 0.190)
Yes	107 (21)	27 (15)	80 (25)		
No	394 (79)	150 (85)	244 (75)		

a Independent samples *t* test; Pearson χ2 test; Fisher exact test.

b Effect size measures: Cohen *d* [95% CI] for continuous outcomes [15]; Cramer *V* [16,17] [95% CI] for categorical outcomes [18-20].

c GED: general educational development certificate (high school equivalent).

### Recruitment

#### Participant Flow

**Figure 2.**
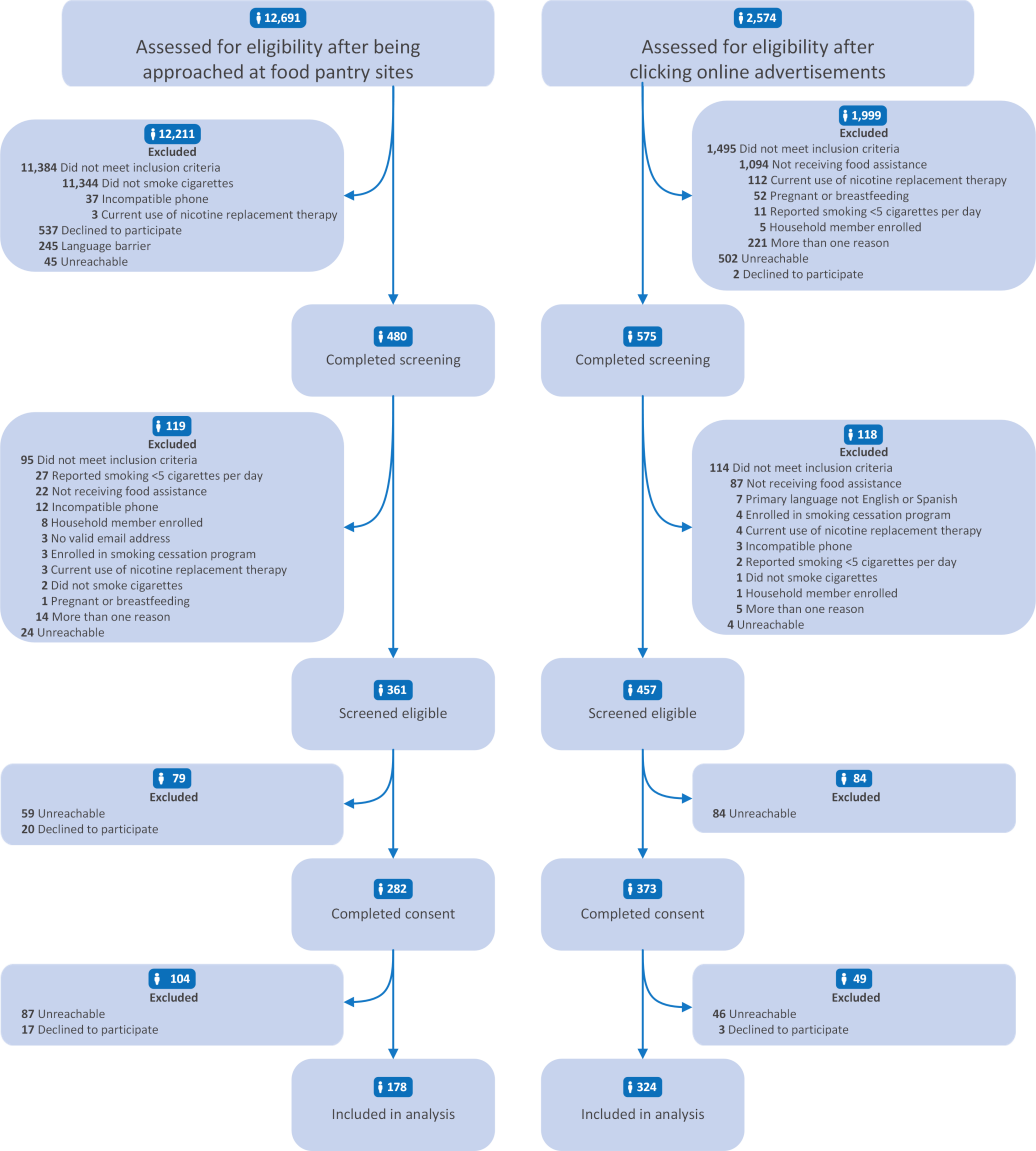
Secondary analysis of data from a mobile health smoking cessation randomized controlled trial for people with food insecurity: CONSORT-style flow diagram by recruitment modality. CONSORT: Consolidated Standards of Reporting Trials.

As detailed in Figure 2, the large majority of potential recruits via both in-person and online modalities were excluded via prescreening before completing the eligibility screener. Of 12,691 approaches at food pantries, 12,211 (96%) did not complete the eligibility screener, predominantly because they did not currently smoke cigarettes (11,344/12,211, 93%). Potential online recruits were excluded at a lower rate of 78% (1999/2574 total potential online recruits); most were either not receiving food assistance (1094/1999, 55%) or were unreachable (502/1999, 25%).

#### Intervention and Comparator Delivery

Site visits yielded 178 participants over 23 months, an in-person enrollment rate of 7.7 per month. Online recruitment garnered 324 participants over 5 months—an enrollment rate of 64.8 per month—resulting in a final sample size of 502. A total of 1055 individuals (online: 575, 55% and in person: 480, 45%) completed the eligibility screener. Potential online and in-person recruits did not differ significantly on study eligibility (457/575, 79%) versus (361/480, 75%; OR 1.27, 95% CI 0.95-1.72; *χ*^2^_1_=2.7; *P*=.10). However, eligible individuals recruited online were more likely to enroll (324/457, 71%) than eligible individuals recruited in person (178/361, 49%; OR 2.50, 95% CI 1.86-3.38; *χ*^2^_1_=39.7; *P*<.001). Among those who completed the eligibility screener, enrollment was higher for online recruits (324/575, 56%) than for in-person recruits (178/480, 37%; OR 2.19, 95% CI 1.70-2.83; *χ*^2^_1_=38.9; *P*<.001). Thus, recruitment modality was primarily associated with enrollment among eligible participants, rather than eligibility itself.

Online advertisement impression data were not available. A total of 2574 individuals clicked an advertisement for the study. Among those who clicked an advertisement, 12.6% (324/2574) enrolled in the trial. In contrast, 1.4% (178/12,691) of all individuals approached in person ultimately enrolled; however, enrollment was 13.2% (178/1347) among those not immediately ruled out at prescreening due to self-reported nonsmoking (12,691 approached minus 11,344 reporting nonsmoking).

The onboarding completion rate (ie, full enrollment) among those who completed consent was higher for online recruits (324/373, 86.9%) than for in-person recruits (178/282, 63.1%). Enrollment rates among those who both completed consent and the baseline questionnaire were 97.0% (324/334) for online recruits and 89.4% (178/199) for in-person recruits. Screening to enrollment times were shorter and less variable for online recruits (median 1, IQR 0-2 d; and mean 2.2, SD 5.1 d) than for in-person recruits (median 2, IQR 1-8 d; mean 10.0, SD 39.6 d). Similarly, fewer contact attempts were made prior to enrollment for online recruits (median 0, IQR 0-1; and mean 0.5, SD 0.9) than for in-person recruits (median 1, IQR 1-3; and mean 2.5, SD 3.1).

### Harms

No adverse events were reported, nor were any stopping rules triggered during the trial.

### Ancillary Analyses

Little’s MCAR test indicated that the data used in the exploratory hierarchical linear regression analysis were not MCAR (*χ*^2^_32_=52.9; *P*=.01). To evaluate the sensitivity of the results to the handling of missing data, multiple imputation by chained equations was conducted, generating 20 imputed datasets using predictive mean matching [30]. The pooled regression results using the multiply-imputed datasets did not differ substantively on predictor significance or effect magnitude from those obtained using listwise deletion, and conclusions were unchanged. Thus, for parsimony and ease of interpretation, results presented below are based on the complete-case analyses.

Exploratory hierarchical linear regression analysis results (Table 2) indicated that, as a set, the demographic covariates explained a significant proportion of the variance in self-reported food insecurity (*R*^2^=0.098, 95% CI 0.040-0.136; *F*_7,467_=7.21; *P*<.001). When recruitment modality was added as a predictor, the resulting model (ie, model 2) remained significant (*R*^2^=0.150, 95% CI 0.081-0.194; *F*_8,466_=10.29; *P*<.001); the improvement in model fit was significant (*F*_1,466_=28.86; *P*<.001). Critically, recruitment modality accounted for approximately an additional 50% of the variability in self-reported food insecurity above and beyond the demographic factors alone (Δ*R*^2^=0.053; percentile bootstrapped 95% CI 0.019-0.100; BCa bootstrapped 95% CI 0.020-0.101). Briefly, in model 2, age (*b*=−0.03, 95% CI −0.05 to −0.01; *P*=.003) and having an annual household income ≥$20,000 (*b*=−0.52, 95% CI −0.91 to −0.14; *P*=.008) were significantly associated with lesser food insecurity. In contrast, female sex (*b*=0.49, 95% CI 0.06-0.92; *P*=.03) and being recruited online (vs in person; *b*=1.22, 95% CI 0.78-1.67; *P*<.001) were significantly associated with greater food insecurity.

**Table 2.** Secondary analysis of data from a mobile health smoking cessation randomized controlled trial for people with food insecurity: predicting food insecurity (0‐6) with associated demographic variables (model 1) versus demographic variables plus recruitment modality (model 2).

Predictor	Model 1^a^ (n=475^b^)		Model 2^c^ (n=475^b^)^d^	
b (95% CI)	*P* value	b (95% CI)	*P* value
Age (y)	−0.05 (−0.06 to −0.03)	<.001	−0.03 (−0.05 to −0.01)	.003
Non-Hispanic White	0.61 (0.20 to 1.03)	.004	0.24 (−0.19 to 0.67)	.27
Female	0.56 (0.12 to 1.00)	.01	0.49 (0.06 to 0.92)	.03
LGBT+^e^	0.26 (−0.31 to 0.83)	.37	0.14 (−0.42 to 0.69)	.63
Educated beyond HS^f^ or GED^g^	0.47 (0.07 to 0.88)	.02	0.26 (−0.15 to 0.66)	.21
No health insurance coverage	0.23 (−0.30 to 0.75)	.40	0.38 (−0.13 to 0.89)	.15
Income of US $20,000 or more per year	−0.54 (−0.94 to −0.15)	.007	−0.52 (−0.91 to −0.14)	.008
Recruited online (vs in person)	—^h^	—	1.22 (0.78 to 1.67)	<.001

a *R*2: b=0.098, 95% CI 0.040-0.136.

b Cases with any missing predictor data (n=27) were deleted listwise, yielding the analytic sample of 475 participants.

c *R*2: b=0.150, 95% CI 0.081-0.194.

d Change in model fit: *F*_1, 466_=28.86; *P*<.001; Δ*R*2=0.053, 95% CI 0.019-0.100.

e LGBT+: lesbian, gay, bisexual, transgender, and others.

f HS:high school.

g GED: General educational development certificate (high school equivalent).

h Not applicable.

## Discussion

### Interpretation

This secondary analysis examined differences in baseline sociodemographic, psychosocial, and smoking-related characteristics of participants recruited in person and online to a smoking cessation RCT for people with food insecurity and compared differences in recruitment pace and enrollment throughput across in-person versus online recruitment methods. Results indicated that participants recruited in person at food distribution events in West Central Florida differed in important and sometimes paradoxical ways from those recruited online via social media. Given our use of these 2 different modalities at different points in the study, a secondary objective was to examine the relative speed of in-person versus online recruitment. Following relatively slow in-person recruitment, switching to an online, national recruitment strategy was associated with a substantially higher enrollment rate; however, other concurrent differences between the strategies and their consequences, such as how in-person and online recruits became aware of the study (ie, face-to-face interactions with study personnel vs online via advertisements on social media or internet search results) and geographical reach (regional vs national) are important to consider.

To our knowledge, this study provided the most comprehensive comparison of the personal characteristics of participants recruited via different modalities to a smoking cessation RCT for a special population of any kind. The comparison revealed noteworthy sociodemographic similarities between groups, including relationship status, living in a household with another person who smoked, and employment status. However, there were also important group differences. In-person recruits had lower household income and educational attainment. They were also significantly older, more ethnically diverse, and more likely to be male. Despite having lower socioeconomic status, participants recruited at in-person food distribution events reported lower levels of loneliness, greater resilience, fewer depressive symptoms, higher subjective social status, and lower levels of perceived financial strain (despite having lower incomes). These protective characteristics [31,32] may have reflected greater engagement in their communities [33] and receiving services from a trusted community organization (ie, Feeding Tampa Bay) [34]. They could also represent self-selection into receiving on-site food assistance services by people who already felt a relatively strong connection to their communities [35]. That said, it remains possible that regular in-person engagement with community-based resources may have engendered a sense of belonging and support [36]. Furthermore, the additional resources to address food insecurity provided by food banks (above and beyond all other possible sources of food aid, such as EBT or food stamps) may have resulted in the in-person recruits having more food available on average than the individuals recruited online, potentially helping to explain the considerable gap in food insecurity between the recruitment groups [37].

It is also notable that relative to online recruits, in-person recruits scored more favorably on a variety of characteristics associated with smoking cessation success. These included considerably higher levels of food security [5], lesser financial strain [38], higher subjective social status [39], better psychosocial functioning [40,41], and greater readiness to quit smoking [42]. An important caveat is that although in-person recruits were less likely to report using recreational drugs, they were more likely to endorse alcohol misuse and more likely to consume noncigarette tobacco products (eg, cigars). In keeping with vaping’s greater frequency among younger cohorts [2], however, in-person recruits reported both having tried vaping and current vaping less frequently than online recruits. Finally, the exploratory hierarchical linear regression analysis suggested that the observed difference in food security between the groups was not solely a byproduct of contrasting basic demographic characteristics. The results were consistent with the possibility that differences in behavioral and psychological characteristics between the groups might help explain the sizable difference in food insecurity observed between the groups. Although the analyses could not establish causal pathways, the in-person and online recruits nonetheless differed considerably in terms of risk and protective factors found to predict successful smoking cessation in previous studies.

On average, individuals recruited online may have been more technologically adept, comfortable with digital platforms, and willing to engage with smartphone-based care than individuals recruited in person, who had not proactively self-selected into screening for participation via a personal electronic device but rather had been approached at a food bank and invited to complete the screener via tablet [43,44]. Comfort and skill with technology might not only be relevant to recruitment outcomes but also might have downstream consequences for intervention uptake, follow-up completion, and cessation outcomes [45,46].

Taken together, findings suggest that a 2-pronged recruitment approach involving both in-person and online methods may be needed to effectively target the wide spectrum of individuals seeking food assistance. It is possible that differences might not have been observed if online recruitment had been conducted only in the same geographic area that in-person recruitment took place [47]. Thus, online recruitment may not always serve as a complete substitute for “boots on the ground” approaches, depending on researchers’ goals and priorities. Furthermore, results from the pilot trial on smoking cessation treatment recruitment at food pantries in Greater Cleveland suggest that in-person recruitment at food distribution events resulted in a slightly higher proportion of people seeking food assistance who enrolled. Nonetheless, the enrollee yield rate in that study was still very modest [11]. Given the divergence in the personal profiles of the in-person and online recruits, it could be beneficial to use stratified or adaptive recruitment methods to improve representation of groups of interest recruited via a given method. For example, recruitment modality quotas could be prespecified or online advertisement spending reallocated toward underrepresented subgroups in real time.

Intriguingly, although participants recruited in person and online were equally likely to screen eligible, the probability of enrolling in the study was much higher for individuals recruited online, suggesting a need for future research designed to carefully evaluate differences in recruitment yield by recruitment strategy. For example, the relatively private, impersonal online approach might encourage some otherwise reluctant individuals to enroll [48], while others might more effectively be persuaded to enroll via in-person interactions with study personnel [49]. Learning which approach is likely to be more effective for different groups of people who smoke could improve both recruitment speed and the reach of future smoking cessation trials and programs.

### Limitations

First, it should be noted that in-person approaches and online advertisement clicks represent different stages of study exposure and intent to participate (staff-initiated initial contact vs self-selected engagement). Thus, comparisons of the percentages of individuals recruited via in-person versus online modalities who enrolled should be contextualized in terms of those differences, although the conversion percentages become more directly comparable later in the enrollment process (eg, enrollment rates among those who provided consent to participate and completed the baseline questionnaire). Moreover, possible geography-based differences between in-person and online recruits included overall rurality, state and local smoking ordinances, and the prevalence of smoking in participants’ immediate environments [47]. Furthermore, online and in-person recruitment occurred during different periods, which might have influenced both receptiveness and observed differences. Future researchers could avoid related concerns by recruiting via both modalities simultaneously and, if feasible and desired, ensuring that online and in-person participants live in the same region (eg, a cancer center’s catchment region).

Contextual priming effects (eg, answering questions in the food bank environment) [50], social desirability bias (eg, wanting to please study staff) [51], and differential modes of survey administration (eg, using personal phones vs tablets provided by study staff to fill out questionnaires) were also possible [43,44]. Nevertheless, mode of survey administration was not systematically recorded, and some participants filled out the baseline questionnaire while at the food bank (albeit in a private area); thus, contextual priming, social desirability, and survey administration mode–based effects cannot be conclusively ruled out as alternative explanations for observed psychological differences between recruitment groups. Future researchers could record device type and administration context (eg, on-site with staff administration, on-site without staff administration, or offsite) to directly test for such effects.

Finally, given the large sample size involved and the number of comparisons conducted, some significant differences might have been of limited practical importance [52] or due to random chance [53]. On the other hand, key differences between the groups (eg, age) had effect size CIs indicating that the magnitude of the group difference was medium to large, in addition to having very low *P* values, and the FDR-adjusted analyses for Table 1 suggested that nearly all significant results were not chance occurrences resulting from having conducted multiple comparisons.

### Conclusions

Prior to this secondary analysis, differences in the baseline characteristics of participants enrolled in person versus online, and differences in recruitment pace across methods, had not been examined in a smoking cessation RCT for people with food insecurity. Nonetheless, it is important to stress that this study is a descriptive comparison within a specific trial context, rather than strong evidence for the comparative advantage of one recruitment modality over another. Regardless, using both recruitment strategies when conducting smoking cessation intervention trials for people with food insecurity may broaden reach, although, as seen in this RCT, the pace of enrollment might differ considerably between the 2 approaches. Additionally, the use of both online and in-person strategies might yield a sample that more closely reflects the underlying population of individuals who smoke and are food insecure. This study expands the RCT recruitment strategy evidence base by providing concrete results to inform researchers designing and conducting smoking cessation trials for special populations, particularly people with food insecurity. Furthermore, examining whether treatment effects and follow-up assessment completion status are influenced by recruitment modality will be important to understanding the potential substantive implications of recruiting from this population via online versus in-person methods.

## Supplementary material

10.2196/80530 Multimedia Appendix 1 R Code for obtaining 95% CI for ΔR^2^.

10.2196/80530 Checklist 1 CONSORT 2025 checklist.

10.2196/80530 Checklist 2 CONSORT E-HEALTH checklist.

## Abbreviations

AT: automated treatment
BCa: bias corrected and accelerated
CONSORT: Consolidated Standards of Reporting Trials
CONSORT-EHEALTH: Consolidated Standards of Reporting Trials of Electronic and Mobile Health Applications and Online Telehealth
EBT: electronic benefits transfer
FDR: false discovery rate
MCAR: missing completely at random
OR: odds ratio
RCT: randomized controlled trial
REDCap: Research Electronic Data Capture
ST: standard treatment
