# Supplementary material for: Sociodemographic Paradoxes and Enrollment Differences in In-Person Versus Online Recruitment to a Mobile Health Smoking Cessation Intervention for Food-Insecure Adults: Secondary Analysis of a Randomized Controlled Trial
Source: J Med Internet Res. 2026 Jun 11;28:e80530. doi: 10.2196/80530 (PMC13258061; doi:10.2196/80530)
Supplement: Multimedia Appendix 1 [file jmir-v28-e80530-s001.docx]

Online Supplement: R Code for Obtaining 95% CI for Δ*R*^2^

#STEP 1: Obtain point estimates for R2 for Model 1 and Model 2 and the #point estimate for delta R2

#First, use base R’s “lm” function to obtain and save the results of #your two models

#Load necessary libraries

library(MBESS)

library(boot)

#Get the R2 and N from the first linear regression model

summary_m1 <- summary(model1_exploratory_hlr)

r2_val_m1 <- summary_m1$r.squared

n_size_m1 <- nrow(model1_exploratory_hlr$model) #Total N

p_vars_m1 <- summary_m1$df[1] #Number of predictors

#Calculate the CI

ci.R2_m1 <- ci.R2(R2 = r2_val_m1, N = n_size_m1, p = p_vars_m1, Random.Predictors = FALSE, conf.level = 0.95)

r2_val_m1; ci.R2_m1 #R2 and 95% CI

#Get the R2 and N from the second linear regression model

summary_m2 <- summary(model2_exploratory_hlr)

r2_val_m2 <- summary_m2$r.squared

n_size_m2 <- nrow(model1_exploratory_hlr$model) #Total N

p_vars_m2 <- summary_m2$df[1] #Number of predictors

# Calculate the CI

ci.R2_m2 <- ci.R2(R2 = r2_val_m2, N = n_size_m2, p = p_vars_m2, Random.Predictors = FALSE, conf.level = 0.95)

r2_val_m2; ci.R2_m2 #R2 and 95% CI

#Obtain the point estimate for delta R2:

r2_val_m2 - r2_val_m1 #Subtract R2 for Model 1 from R2 for Model 2

#STEP 2: Obtain the 95% CI for delta R2

# Define a custom function to use within boot::boot to calculate delta #R2's 95% CI

#Fundamentally, it's as if one is running the study repeatedly using #random sampling with replacement from the observed data (i.e., it is #a nonparametric bootstrapping procedure)

get_delta_r2 <- function(data, indices) {

#"data" is a placeholder for the dataset to be used.

d <- data[indices, ] #When boot::boot (shown later) runs and calls #this function, it generates a list of random row numbers (with #replacement). It passes those numbers into data[indices, ] so #boot::boot knows which specific randomly sampled rows to use for this #specific iteration.

fit1 <- lm(model1_exploratory_hlr, data = d) #Model 1's results were #saved as "model1_exploratory_hlr"

fit2 <- lm(model2_exploratory_hlr, data = d) #Model 2's results were #saved as "model2_exploratory_hlr"

return(summary(fit2)$r.squared - summary(fit1)$r.squared) #Returning #the delta R2 for this iteration

}

#Run the bootstrapping procedure

set.seed(xxxx) #Set seed to an integer value of your choice to ensure #repeatability of results

boot_results <- boot::boot(data = nrm2, statistic = get_delta_r2, R = 10000) #May take several minutes

hist(boot_results) #Especially if histogram of the delta R2 values is #skewed, advisable to obtain and report both a percentile and a bias-#corrected and accelerated (or BCa) version of the 95% CI

#Get the percentile CI

boot::boot.ci(boot_results, type = "perc")

#Get the BCa CI

boot::boot.ci(boot_results, type = "bca")
